# Advances in Hydrogels for Meniscus Tissue Engineering: A Focus on Biomaterials, Crosslinking, Therapeutic Additives

**DOI:** 10.3390/gels10020114

**Published:** 2024-02-01

**Authors:** Zhuxing Zhou, Jiajie Wang, Chaoqian Jiang, Kaiwang Xu, Tengjing Xu, Xinning Yu, Jinghua Fang, Yanyu Yang, Xuesong Dai

**Affiliations:** 1 Department of Orthopedic Surgery, the Second Affiliated Hospital, Zhejiang University School of Medicine, Hangzhou 310000, China; 2 Orthopedics Research Institute of Zhejiang University, Hangzhou 310000, China; 3 Key Laboratory of Motor System Disease Research and Precision Therapy of Zhejiang Province, Hangzhou 310000, China; 4 Clinical Research Center of Motor System Disease of Zhejiang Province, Hangzhou 310000, China; 5 School of Materials and Engineering, Zhengzhou University, Zhengzhou 450001, China

**Keywords:** hydrogel, biomaterial, crosslinking, therapeutic effect, meniscus, repair, regeneration, tissue engineering

## Abstract

Meniscus tissue engineering (MTE) has emerged as a promising strategy for meniscus repair and regeneration. As versatile platforms, hydrogels have gained significant attention in this field, as they possess tunable properties that allow them to mimic native extracellular matrices and provide a suitable microenvironment. Additionally, hydrogels can be minimally invasively injected and can be adjusted to match the shape of the implant site. They can conveniently and effectively deliver bioactive additives and demonstrate good compatibility with other functional materials. These inherent qualities have made hydrogel a promising candidate for therapeutic approaches in meniscus repair and regeneration. This article provides a comprehensive review of the advancements made in the research on hydrogel application for meniscus tissue engineering. Firstly, the biomaterials and crosslinking strategies used in the formation of hydrogels are summarized and analyzed. Subsequently, the role of therapeutic additives, including cells, growth factors, and other active products, in facilitating meniscus repair and regeneration is thoroughly discussed. Furthermore, we summarize the key issues for designing hydrogels used in MTE. Finally, we conclude with the current challenges encountered by hydrogel applications and suggest potential solutions for addressing these challenges in the field of MTE. We hope this review provides a resource for researchers and practitioners interested in this field, thereby facilitating the exploration of new design possibilities.

## 1. Introduction

**Figure 1:**
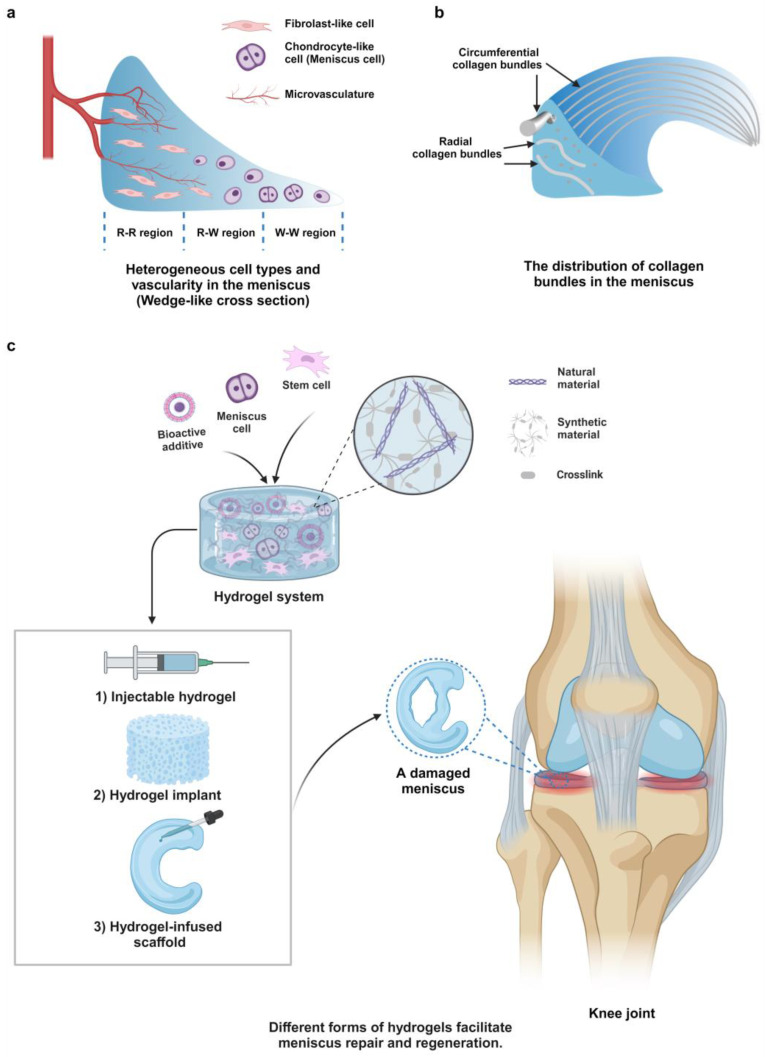
Meniscus structural features and application of different forms of hydrogels in MTE. (**a**) Heterogeneous cell types and vascularity in the meniscus (wedge-like cross section); R-R region: red–red region; R-W region: red–white region; W-W region: white–white region. (**b**) Collagen bundles in the meniscus align in both the circumferential and radial directions. (**c**). The application of different forms of hydrogels in meniscus repair and regeneration. The figure was created with BioRender.com.

The menisci are semilunar fibrocartilages positioned between femoral condyles and tibial plateaus of knee joints [1,2]. They serve as critical components of knee joints because of their pivotal roles in stabilizing joints, lubricating articular cartilages, distributing load stresses, absorbing shocks, and contributing to knee joint proprioception [3,4,5]. The menisci possess irregularly geometric morphology; when observed from the coronal plane, they function as wedge-shaped cushions that fill the spaces between the femur and tibial bones [1,4]. Moreover, these fibrocartilages have typical heterogeneous features, with the structures exhibiting the zone-specific distribution of cell phenotypes and extracellular matrices (ECMs) [4,5,6,7]. Generally, fibroblast-like cells reside in the outer regions of the menisci, whereas chondrocyte-like cells are in the inner regions (Figure 1a) [4]. Correspondingly, ECM compositions mainly contain collagen type I (COL I) in meniscal outer regions; however, collagen type II/COL II) and proteoglycans are mainly distributed in the inner regions [4,5,6,7]. The abundant COL I fibers assemble into collagen bundles mainly aligned in circumferential and radial directions to withstand tensile forces (Figure 1b), whereas COL II molecules aggregate with proteoglycans in the inner regions to resist compressive forces [4,5,6,7].

The morphological and structural features result in the transmission of 50–85% of the compressive load in the knee joints through the menisci [4]. Moreover, the menisci increase the contact area and decrease the contact pressure in the tibiofemoral compartments of the knee joints [8], thereby protecting articular cartilage from degeneration [9]. 

In addition to unique microstructures, the mature menisci also have a region-specific spatial distribution of blood supply, with only a small percentage of blood vessels found in the outer 10–25% of the tissues [4,10]. Based on the distribution of blood vessels, menisci can be classified into three regions (Figure 1a). These regions are referred to as the red–red region, the red–white region, and the white–white region, with the red–red region located in the peripheral rim of the menisci and the white–white region located towards the central rim [5,11]. Because of their low vascularity, menisci have an intrinsic limitation in their capacity to heal themselves [12]. Unfortunately, menisci are susceptible to tearing due to acute injuries or chronic degeneration [3,11], which are the most common orthopedic injuries seen in knee clinics [13]. With the rapid development of surgical techniques, various meniscus repair techniques, such as arthroscopic-assisted meniscus suturing, have been widely adopted for meniscus repair. Despite the significant advances in surgical techniques, not all meniscus tears can be successfully repaired. Indeed, arthroscopic partial meniscectomy (APM) remains the gold standard for managing meniscus tears in the USA [11,14]. This procedure aims to alleviate symptoms and preserve as much healthy meniscus tissue as possible, particularly in cases where the tear is deemed irreparable. However, even a minor partial meniscectomy can have an impact on knee motion kinetics and the biomechanics of the meniscus, increasing the risk of further injury [15]. Recent research has indicated that APM is connected to a higher likelihood of developing knee osteoarthritis, which remains an irreversible disease, causing significant chronic pain and disability [16,17]. Meniscal allograft transplantation (MAT) has been a promising treatment for enhancing knee functionality [18]. Nonetheless, the widespread use of MAT in clinical settings is restricted due to the limited availability of suitable grafts, the challenge with long-term graft survival, and the risk of disease transmission [19,20].

**Figure 2:**
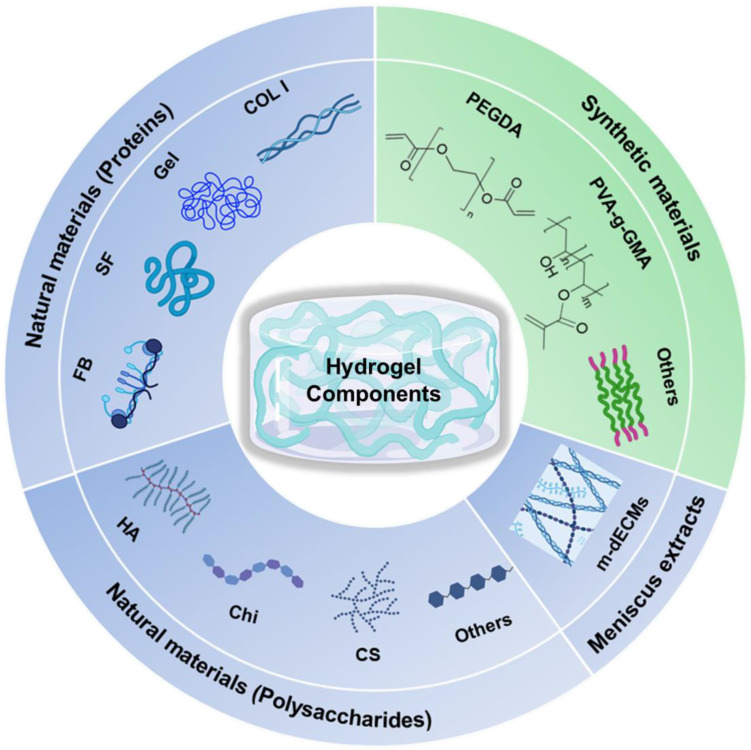
Classification of common materials used for hydrogel construction; natural materials (blue patch). Proteins are summarized in the upper left quadrant, polysaccharides are in the lower left quadrant, meniscus extracts are in the lower right quadrant, and synthetic materials (green patch) are in the upper right quadrant. The figure was created with BioRender.com.

**Figure 3:**
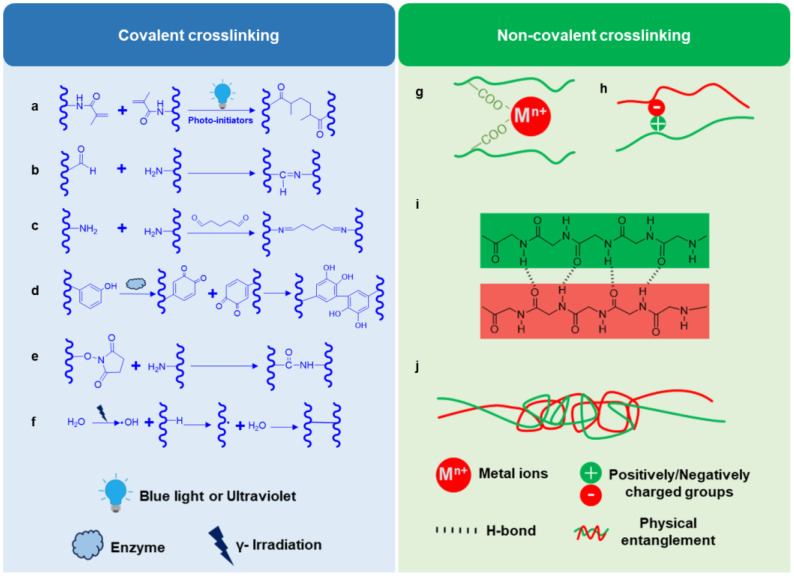
Schematic diagram of common crosslinking strategies for hydrogel formation. Covalent crosslinking strategies are presented in the left panel (blue), including (**a**) photo-crosslinking, (**b**) the Schiff base reaction, (**c**) the use of crosslinking agents, (**d**) enzyme-mediated crosslinking, (**e**) N-hydroxysuccinimide ester (NHS-ester) chemistry, and radiation-induced crosslinking (**f**). Non-covalent crosslinking strategies are summarized in the right panel (green), including (**g**) ionic crosslinking, (**h**) electrostatic interaction, (**i**) hydrogen bonds, and (**j**) physical entanglement.

Recently, there has been a surge in interest regarding the application of tissue-engineering technologies for the repair and regeneration of the meniscus, namely, meniscus tissue engineering (MTE) [21,22,23,24]. This is a promising approach to treating irreparable meniscus injuries and restoring healthy meniscus structures and functions. Seeding cells and/or anchoring growth factors in various biomaterials to construct meniscus scaffolds is a classical paradigm in the field of MTE [11]. For instance, polycaprolactone (PCL) scaffolds fabricated with the electrospinning technique and sponge silk scaffolds fabricated via freeze-drying have been evaluated in MTE and exhibited clinical potential in meniscus reconstruction [25,26]. Three-dimensional (3D) printing has been flourishing as a powerful tool for tissue engineering via the construction of complex 3D biomimetic architectures [27]. In the field of MTE, 3D-printed (3DP) meniscus scaffolds have also been extensively utilized for meniscus repair and regeneration, as 3DP scaffolds can mimic the irregular meniscus structural features from a patient’s medical images, thereby displaying advantages over traditional techniques like the aforementioned electrospinning and freeze-drying [28,29]. Nonetheless, pure 3DP scaffolds are not ideal, as most of them are composed of thermoplastic biomaterials such as PCL, which have some drawbacks. For example, PCL has a hydrophobic nature that hinders cell adhesion [30]. Although PCL is mechanically strong, the pure PCL meniscus scaffold can result in severe wear at the surface of knee cartilage because of its high compressive stiffness [31]. In comparison, hydrogels are hydrophilic and soft 3D polymeric networks that have some unique advantages and have also been widely applied in different forms to facilitate meniscus repair and regeneration (Figure 1c). Hydrogels can mimic the natural ECM biophysical and biochemical properties, such as high water content, appropriate stiffness, and adhesion relative cues, thus providing an ideal microenvironment for cells [32,33,34]. In addition, hydrogels serve as ideal delivery vehicles, capable of loading cells, growth factors (GFs), drugs, and other bioactive additives to enhance therapeutic effects [35,36]. Before gelation, hydrogel precursors feature flowability and easy manipulation, enabling the hydrogels to be easily molded into complex shapes and geometries. For example, injectable hydrogels (Figure 1c-1) have the potential to be easily applied through minimally invasive techniques for meniscus repair, such as injection [37]. Hydrogel implants (Figure 1c-2) can also be constructed rapidly and be transplanted through arthroscopic surgery [37,38]. Despite their potential to heal meniscus injury and fill partial meniscus defects, pure hydrogels often suffer from inadequate mechanical properties compared to native meniscus tissues. As a result, they cannot effectively bear joint loads or absorb shock, which is essential for eligible meniscus substitutes. To overcome weak mechanical properties in pure hydrogels, hydrogel-infused meniscus scaffolds (Figure 1c-3) have been developed and widely explored, where hydrogels are used to modify and improve the performance of other biomaterials, such as 3DP PCL scaffolds [39,40,41,42,43]. The composite scaffolds combine the various aforementioned advantages of hydrogel and strong 3DP backbones, thus having a wide spectrum of applications in MTE. 

The use of hydrogel in MTE has been covered by some recent reviews, which featured discussions on hydrogel application in meniscus repair, rehabilitation, and regeneration [44,45]. By conducting a detailed analysis of the available literature in recent decades, our review aims to discuss hydrogel design, with a focus on biomaterial properties and functions, crosslinking strategies, and therapeutic components loaded by hydrogels, including cells and other bioactive additives. In addition, we analyze and discuss the therapeutic mechanisms offered by these therapeutic components in MTE. We not only provide a comprehensive summary of these aspects but also compare their advantages and disadvantages when used as a member of the hydrogel systems for meniscus repair and regeneration. Based on this, we propose some crucial aspects that are easily overlooked when designing future hydrogel products for MTE use. In conclusion, we review the current status, challenges, and emerging trends in the utilization of hydrogels in MTE. We intend for this review to serve as a useful resource for researchers and practitioners interested in this field, thereby facilitating the exploration of new design possibilities. 

## 2. Biomaterials for Constructing Hydrogels

The components of hydrogels are crucial for determining their properties and applications. Based on the sources of their compositions, they can be categorized into two main types: natural materials and synthetic materials. These materials can be chemically modified to produce a range of derivatives, which possess additional functions and a broader application spectrum. They are summarized in Figure 2 and Table 1. It should be noted that some biomaterials have been shown to have the ability to generate fibrotic tissue phenotype, whereas others promote the formation of cartilage-like phenotype, as summarized in Table 1. To precisely restore the heterogeneous features of the native meniscus, the different biological functions of the biomaterials should be considered when designing hydrogel products. In addition, both natural and synthetic materials have their unique advantages and disadvantages. Therefore, they are often used in combination, leveraging the strengths of each material while mitigating their weaknesses. However, to clarify these material characteristics clearly, we will discuss them individually.

### 2.1. Natural Materials 

Hydrogels composed of natural biomaterials have similar advantages as natural ECMs because the materials are extracted from plants or animals, which possess biocompatibility, biodegradability, and bioactivity, making them suitable for biomedical applications [46]. Commonly used natural materials and their derivatives include proteins (Figure 2; Table 1): collagen type I (COL I) [40,47,48,49,50], gelatin (Gel) [24,51,52] and gelatin methacrylate (GelMA) [42,43,53,54,55], fibrinogen (FB) [38,56,57,58,59]; silk fibroin (SF) [41], polysaccharides (Figure 2; Table 1) such as hyaluronic acid (HA) [47,50] and tyramine-modified hyaluronic acid (TA-HA) [60], alginate (Alg) [61,62,63,64] and alginate dialdehyde (ADA) [51], agarose (Ag) [42,43,65], cellulose [55,61,66], chondroitin sulfate (CS) [67], and chitosan (Chi) [37,68]. Additionally, meniscus extracts (Figure 2; Table 1), also referred to as meniscus decellularized extracellular matrices (m-dECMs) [23,53,62,63,69,70,71,72], have been widely used for constructing hydrogels. 

#### 2.1.1. Collagen Type I (COL I)

COL I is the main ECM component of the native meniscus [5]. It has been reported that COL I supported mesenchymal stem cells (MSCs) towards fibrochondrogenic differentiation [23]. Additionally, COL I can slowly self-polymerize into a hydrogel in a neutral environment and at a temperature of 37 °C [73], making COL I hydrogels useful tools for delivering cells and other bioactive additives. Because of these advantages, COL I serves as an ideal substrate for cell adhesion, proliferation, and differentiation, facilitating the regeneration of meniscus tissue [47,74]. However, pure collagen hydrogel is limited by its mechanical weakness. Some researchers therefore chose to mix cells and COL I and infused them into a polycarbonate urethane (PCU)-based porous scaffold for meniscus regeneration [48]. After 14 days of culture, a greater number of viable cells seeded with collagen had formed significant intercellular bridges compared to the cells on the bare PCU scaffolds. 

**Table 1:** Summary of biomaterials for constructing hydrogels in MTE and their functions.

Categories (Subtypes)	Names (Derivatives)	Functions	References
Natural materials (proteins)	Collagen type I	Stimulating cell proliferation	[40,47,48,49,50]
		Promoting cell adhesion	
		Inducing fibrochondrogenic differentiation of cells	
	Gelatin (GelMA)	Stimulating cell proliferation	[24,43,51,52,53,54,55]
		Promoting cell adhesion	
		Inducing cells to acquire a fibrotic phenotype	
	Silk fibroin	Supporting cell growth and adhesion	[41]
	Fibrinogen	Supporting cell growth and adhesion	[38,56,57,58,59]
Natural materials (polysaccharides)	Hyaluronic acid (TA-HA)	Promoting meniscus cell proliferation and migration Chondrogenesis Inhibiting meniscus cell apoptosis Inflammatory modulation	[47,50,60,75]
	Alginate (ADA)	Supporting tissue regrowth	[51,61,62,63,64]
		Improvement in biocompatibility	
	Agarose	Supporting cell growth Chondrogenesis	[42,43,65]
	Cellulose	Improving hydrogel mechanical properties	[55,61,66]
	Chondroitin sulfate	Promoting cell proliferation	[67]
		Chondrogenesis	
	Chitosan	Supporting cell growth Promoting cell proliferation Potential chondrogenesis	[37,68,76]
Natural materials (meniscus extracts)	m-dECMs	Supporting cell growth Promoting cell proliferation Sustaining meniscus cell phenotype Inducing fibrochondrogenic differentiation of cells	[23,53,62,63,69,70,71,72,77]
Synthetic materials	PEG (PEG-CHO, PEG-NHS, PEG-NH_2_, PEGDA)	Supporting cell growth Encapsulating cells and additives	[37,39,61,78]
	PVA (PVA-g-GMA)	Supporting cell growth Improving hydrogel strength	[66]
	F-127 and PEO (F127DA)	Supporting cell growth Encapsulating cells and additives Improving hydrogel strength	[55,56,57]
	KI24RGDS	Supporting cell growth Stimulating cell proliferation Promoting cell adhesion	[79]

GelMA: gelatin methacrylate; TA-HA: tyramine-modified hyaluronic acid; ADA: alginate dialdehyde; m-dECMs: meniscus-decellularized extracellular matrices; PEG: polyethylene glycol; PEGDA: PEG dialdehyde; PVA: poly (vinyl alcohol); PVA-g-GMA: PVA-grafted glycidyl methacrylate; F-127: Pluronic^®^ F-127; PEO: polyethylene oxide; F127DA: FA127 diacrylate; KI24RGDS: a synthetic peptide with lysine (K), isoleucine (I), arginine (R), glycine (G), aspartic acid (D), and serine (S).

#### 2.1.2. Gelatin (Gel)

Gelatin hydrolyzed from collagen has better solubility and less antigenicity than collagen, which contains many arginine–glycine–aspartic acid (RGD) sequences, enhancing cell adhesion, proliferation, and a series of ensuing cascades [80]. As a type of cold-setting hydrogel, gelatin is thermally reversible, exhibiting a classical sol-gel transition as the surrounding temperature changes [81]. Moreover, gelatin can be easily subjected to chemical modification or physical gelation [82]. For example, gelatin methacrylate (GelMA) is an exceptional biocompatible biomaterial that is derived from gelatin through partial methacrylation [80]. It is created by chemically modifying gelatin using methacrylic anhydride or other reactive methacrylates [80]. GelMA not only retains the favorable biocompatibility and thermal response characteristics of gelatin but also possesses the ability of photo-crosslinking [83]. In the presence of initiators, GelMA solutions can be easily crosslinked and rapidly cured into hydrogels by blue light or ultraviolet (UV) irradiation [83]. GelMA hydrogel, once cured, will no longer experience the sol-gel transition as a result of temperature changes. This property makes GelMA precursors ideal for delivering cells into meniscus defects. These cells can be anchored in the locations of the defect through the subsequent curing process, promoting tissue repair [54]. Cell-laden GelMA can also be infused into 3DP scaffolds to form composite meniscus scaffolds for meniscus regeneration [43]. It has been observed that human fibrochondrocytes in GelMA hydrogels exhibit a significantly high level of COL I mRNA, indicating that GelMA facilitates cells to adopt the fibrotic phenotype [43]. 

#### 2.1.3. Fibrinogen (FB)

FB is a natural protein in the blood that plays a crucial role in the coagulation cascade to stop bleeding. The exceptional biocompatibility and cell interaction of this protein renders it a remarkably powerful precursor for the synthesis of diverse biomaterials [84]. FB can be crosslinked by thrombin, which catalyzes the formation of FB-based hydrogels through covalent intermolecular crosslinking [85]. The hydrogels have shown promising potential for delivering cells and other additives in MTE [38,56,57,58,59]. FB is mechanically weak, but it can be incorporated with other robust materials to compensate for the drawback [56,57]. 

#### 2.1.4. Silk Fibroin (SF)

SF is a biomaterial derived from silkworms that has been widely studied and approved for use in biomedical applications by the U.S. Food and Drug Administration (FDA) [86]. Similar to other natural polymers, SF exhibits excellent biocompatibility and biodegradability. It can support cell attachment, growth, and proliferation, which makes it a promising biomaterial for MTE [87,88]. SF-based hydrogels can be prepared by inducing the β-sheet formation with various chemical reagents such as alcohols and via physical methods such as γ-rays [41]. 

#### 2.1.5. Hyaluronic Acid (HA)

HA is a naturally occurring polysaccharide and a critical component found in ECM that has excellent biocompatibility and low immunogenicity [89,90]. The present findings demonstrate that HA has the potential to regulate inflammation and actively participate in extracellular matrix (ECM) remodeling, cell proliferation, and chondrogenesis, which is achieved through its interaction with the cell-surface receptor cluster of differentiation 44 (CD44) [89]. Also, it has displayed its potential in meniscus regeneration by promoting meniscus cell migration, accelerating cell proliferation, and inhibiting cell apoptosis through the phosphoinositide 3-kinase (PI3K)/mitogen-activated protein kinase (MAPK) pathway via CD44 receptor-mediated signal transduction [75]. However, HA cannot form a hydrogel on its own and thus requires additional chemical modification. Notably, in a study conducted by Kim et al., they modified HA using tyramine (TA) to create TA-HA hydrogel for meniscus repair [60]. TA-HA can be crosslinked under mild conditions using tyrosinase catalysis. This process not only imparts tissue-adhesive properties to TA-HA but also demonstrates good biocompatibility with meniscus cells. Consequently, it enhances the matrix synthesis and expression of cartilage-specific genes, including COL I, COL II, and aggrecan. 

#### 2.1.6. Alginate (Alg)

Alg is a polysaccharide derived from seaweed consisting of two types of copolymers, α-L-guluronate (G block) and β-D-mannuronate (M block), which are connected by 1,4-glycosidic bonds [91]. The G block in alginate is capable of forming reversible ion crosslinking with divalent cations like calcium ions (Ca^2+^), leading to gelation [91]. Alginate-based hydrogels are widely used in the field of tissue engineering. For instance, cell-loaded alginate microgels have the potential to be utilized as injectable engineered scaffolds that facilitate tissue regrowth [92]. However, a limitation of commercially available Alg is the high presence of endotoxins, which poses a safety concern and limits its clinical application [64]. In a recent study by Kim et al., they developed an injectable hydrogel using ultra-purified alginate with low levels of endotoxins [64]. They demonstrated that this hydrogel could promote spontaneous repair of fibrocartilage tissues in a cylindrical meniscal defect model in rabbits. Furthermore, Alg does not possess recognition sites for cell adhesion, which hinders its performance in tissue engineering. To address this limitation, incorporating other protein-based biomaterials such as gelatin could enhance its performance in the field [51].

#### 2.1.7. Agarose (Ag)

Ag is another common polysaccharide extracted from seaweed that has been widely used for tissue engineering purposes. It has remarkable biocompatibility, thermo-reversible gelation behavior, and tunable physiochemical properties, contributing to its suitability as a biomaterial for supporting cell growth and facilitating controlled or localized drug administration [91,93]. Bahcecioglu et al. used Ag to encapsulate meniscus cells to form cell-loaded hydrogels and observed that meniscus cells cultured in agarose demonstrated decreased cell adhesion and rounded cell morphology [65]. Furthermore, these cells produced significantly higher levels of glycosaminoglycans, suggesting that Ag has chondrogenic properties. As a result, agarose may be a suitable material for regenerating the inner region tissues in the menisci.

#### 2.1.8. Cellulose 

Cellulose, composed of β-1,4-linked glucose units, is cheap, eco-friendly, and the most abundant natural polysaccharide on this planet [94]. Cotton-derived cellulose has been approved by the U.S. FDA for medical use due to its higher degree of biocompatibility and non-cytotoxic nature [94]. Furthermore, due to its repetitive building blocks that create highly organized structures, cellulose displays impressive mechanical strength and flexibility [95]. These characteristics make it an ideal material for use as a support structure in hydrogel systems. The incorporation of cellulose nanofibers has substantially improved the mechanical properties of injectable hydrogels, offering great potential for applications in MTE [55,66] considering the vital importance of mechanical strength in meniscus scaffolds.

#### 2.1.9. Chondroitin Sulfate (CS)

CS is an anionic glycosaminoglycan that is another major ECM component in meniscus tissues [5,96]. It plays a critical role in maintaining the structural and functional integrity of healthy meniscus [5]. Due to its non-toxic, biodegradable, and biocompatible properties, it has been widely used in drug delivery and tissue-engineering applications [96]. Additionally, CS has been shown to stimulate the formation of new cartilage by facilitating chondrogenesis [46]. Researchers have utilized n-hydroxysuccinimide (NHS) to modify CS and create injectable CS-NHS biomaterials, possessing favorable biocompatibility and adhesive properties [67]. The CS-NHS formed hydrogels by binding bone marrow and meniscus cells, which have proven to be effective in promoting meniscus repair and regeneration [67]. 

#### 2.1.10. Chitosan (Chi)

Chi is a naturally linear polymer derived from the chitin shells of crustaceans such as shrimp [97]. The chemical structure of chitosan is characterized by the presence of free amino groups on the glucosamine residues, which makes Chi the only natural cationic polysaccharide [98]. Chi has been observed to possess not only excellent biocompatibility but also various beneficial biological properties, such as anti-inflammatory, antibacterial, and antioxidant activity [46]. Furthermore, researchers have discovered that chitosan can create insoluble complexes with collagen and glycosaminoglycans (GAGs) that enhance the process of meniscal regeneration [76,99]. These attributes make it an attractive biomaterial for use in MTE. One notable example is the development of a thermo-responsive hydrogel by combining Chi with β-glycerophosphate sodium (β-GP). This hydrogel is injectable and undergoes a sol-gel transformation from room temperature to 37 °C in the body. It has proven to be highly effective in delivering cells and other drugs for tissue repair and regeneration [100,101]. Canciani and colleagues utilized this hydrogel to load MSCs and noted that it effectively promoted cell proliferation and viability during two weeks of in vitro culture [68]. Furthermore, they implanted the hydrogels containing the cells into the dorsal pouches of nude mice. Specifically, there was a 30% increase in the number of cells at the 12-week mark in vivo compared to a minimal amount observed at the 4-week mark [68]. These findings highlight the potential of this hydrogel in the field of MTE.

#### 2.1.11. Meniscus Decellularized Extracellular Matrices (m-dECMs)

m-dECMs are derived from native meniscus tissues using decellularization techniques, which remove immunogenic cellular components while preserving bioactive molecules and proteins such as collagen, glycosaminoglycans (GAGs), and growth factors [102]. This allows m-dECMs to replicate the microenvironment found in native meniscus tissues, support cell growth, and stimulate cell differentiation [12]. As a result, m-dECMs have been widely utilized for meniscus regeneration [12,23,54,70,72,77,103,104]. Additionally, due to retaining collagen, they can self-assemble into injectable hydrogels or other hydrogel vehicles that carry cells and other bioactive factors for meniscus repair and regeneration [23,40,62,63,70,71].

### 2.2. Synthetic Materials

Synthetic biomaterials are also commonly adopted in the construction of hydrogels in MTE. In comparison with natural biomaterials, synthetic ones provide better options for chemical modifications and molecular changes, allowing for customization of the material properties based on specific application needs [30]. These synthetic biomaterials mainly involve polymers such as polyethylene glycol (PEG), poly (vinyl alcohol) (PVA), poloxamer (Pluronic^®^ F-127), and their derivatives (Figure 2; Table 1). In addition, nature-inspired synthetic peptide hydrogels such as RGD-like peptide hydrogels are intrinsically biocompatible and highly reminiscent of the extracellular matrix (ECM), making them suitable as 3D scaffold materials [105]. It should be noted that some synthetic materials are intrinsically bioinert due to lacking cell affinity sites. To improve their performance, natural materials are frequently incorporated to form composite hydrogels for final use.

#### 2.2.1. Polyethylene Glycol (PEG)

PEG is a biomaterial that has been extensively used in tissue engineering and drug delivery applications due to its biocompatibility, lack of immunogenicity, and resistance to fouling [46,106]. PEG possesses hydroxyl groups at its ends, making it highly hydrophilic and allowing for conjugation with other functional groups [46] such as aldehyde, amino, or diacrylate. For example, PEG diacrylate (PEGDA) is a suitable option for quick and easy injection, as it can swiftly undergo photo-crosslinking to create biocompatible hydrogels. This property makes them highly advantageous for the delivery of cells and/or other additives to facilitate meniscus repair [54,77]. Chen et al. developed an injectable hydrogel based on PEG-aldehyde and chitosan to transport stem cells and growth factors for the healing of a partial meniscus defect [37].

#### 2.2.2. Poly (Vinyl Alcohol) (PVA)

PVA is a semi-crystalline synthetic polymer possessing excellent properties such as biodegradability, biocompatibility, non-toxicity, and low cost [107]. Therefore, it has been reported to be used in multiple biomedical applications such as hydrogel scaffold, wound dressing, and drug delivery [46,108]. PVA also contains hydroxyl groups in its repeating units, which can be readily modified to create PVA derivatives. A recent study conducted by Sinna et al. synthesized PVA-grafted glycidyl methacrylate (PVA-g-GMA) with photo-crosslinking ability [66]. They developed an injectable hydrogel containing 10% PVA-g-GMA and 0.7% cellulose nanofiber (CNF) for meniscus repair. This hydrogel exhibited good biocompatibility, enhancing cell proliferation in vitro and strong mechanical properties after photo-crosslinking and displaying potential for MTE application.

#### 2.2.3. Poloxamer (Pluronic^®^ F-127) and Polyethylene Oxide (PEO)

Pluronic^®^ F-127 (F-127) is a commercially available synthetic material that is composed of amphiphilic blocks of copolymers, consisting of one hydrophobic residue of polypropylene oxide between two hydrophilic units of polyethylene oxide (PEO) [46,109]. F-127 is known for its safety, stability, and ability to form hydrogels at low concentrations at body temperature [110]. Due to its favorable characteristics, F127 is frequently employed as a substrate for injectable drug delivery. Additionally, F127 and PEO can be integrated into other materials to create injectable hydrogels with enhanced mechanical properties for applications in meniscus repair [56,57]. F127 can be conjugated with other active groups via chemical modification. For example, Jeencham et al. used F-127 diacrylate to create photo-crosslinked hydrogels with GelMA and CNF [55]. The resultant combination exhibited encouraging physicochemical and biological characteristics, indicating its potential for use in meniscus repair.

#### 2.2.4. Synthetic Peptide

Okuno et al. recently published a study on an injectable hydrogel composed of a synthetic peptide known as KI24RGDS [79]. The acronym KI24RGDS represents the amino acids lysine (K), isoleucine (I), arginine (R), glycine (G), aspartic acid (D), and serine (S). The peptide is a β-hairpin structure with the amino acid sequence RGDS, which promotes cell adhesion. Additionally, the peptide possesses alternating hydrophilic and hydrophobic amino acid residues, allowing for self-assembly into nanofibers and the formation of hydrogel scaffolds with high stiffness. KI24RGDS exhibits excellent biocompatibility and biodegradability, and its viscosity is sufficient to maintain its position at the site of a meniscal defect following injection. The authors conducted experiments in a rabbit meniscal defect model, which confirmed that the KI24RGDS scaffold effectively facilitated meniscus repair and regeneration.

### 2.3. Pros and Cons

The utilization of natural materials in the formation of hydrogels in meniscus tissue engineering holds significant promise due to their inherent biocompatibility and biodegradability. These properties make them an excellent candidate for supporting tissue regeneration. Moreover, they have some beneficial biological functions, including promoting cell proliferation, fibrochondrogenic/chondrogenic differentiation, antioxidant properties, antiinflammation, and so on [46,47,75]. However, they do face challenges such as batch-to-batch variability, complex structures, potential immunogenicity, and inferior mechanical properties [46]. To address the inherent limitations of natural materials, appropriate chemical modifications serve as a viable approach to impart additional functionality and enhance their physicochemical tunability. Taking GelMA as an example, the introduction of methacryloyl groups allows for precise control over the stability and degradation behavior of the GelMA hydrogel. Furthermore, this modification also enables precise adjustments to its mechanical properties, providing greater flexibility and tunability in its application. In comparison, synthetic materials utilized in hydrogel formation offer other advantages such as stability, customizability, ease of modification, and enhanced mechanical properties [111,112]. However, there are also drawbacks to consider—in particular, potential issues with biological inertness, the slow biodegradation rate, and the generation of toxic degradation products [46]. Blending natural materials into synthetic materials offers a straightforward approach to enhancing their biological activity [56,57]. Furthermore, modifying biologically inert synthetic materials as by grafting RGD peptides serves to augment the material’s affinity with cells, thereby fostering improved interaction and integration within biological systems. Therefore, it is crucial to carefully weigh these factors when selecting and utilizing biomaterials or constructing hydrogels. Overcoming the limitations and optimizing the use of biomaterials will continue to be an area of research in the field of MTE.

## 3. Hydrogel Crosslinking Strategies

Crosslinking plays a crucial role in the formation of hydrogels, as it significantly influences the mechanical and biochemical properties of hydrogels [113]. The crosslinking mechanisms required to form hydrogels vary depending on the specific functional groups interacting between the components [114]. Based on the nature of the chemical bonds involved in crosslinking, these strategies can be categorized into covalent or non-covalent crosslinking [114] (Figure 3). In this section, we focus on the most commonly utilized crosslinking strategies for constructing hydrogels in MTE. It is crucial to highlight that diverse crosslinking strategies are often employed simultaneously within a single hydrogel system. To clarify, each crosslinking strategy will be introduced individually.

### 3.1. Covalent Crosslinking

Covalent crosslinking normally forms strong linkages among polymers in hydrogels [115]. Various methods are used to form covalent crosslinks, including photo-crosslinking (Figure 3a), the Schiff base reaction (Figure 3b), the use of crosslinking agents (Figure 3c), enzyme-mediated crosslinking (Figure 3d), N-hydroxysuccinimide ester (NHS-ester) chemistry (Figure 3e), and radiation-induced crosslinking (Figure 3f).

#### 3.1.1. Photo-Crosslinking

Photo-crosslinking is a commonly employed technique for creating hydrogels in the field of MTE [54,55,66]. The gelation mechanism involves the use of free radicals generated by photo-initiators in response to visible light or ultraviolet radiation, which then initiates the polymerization of hydrogel components containing carbon–carbon double bonds [116]. Photo-crosslinking enables precise control over the formation of hydrogel and its network properties by adjusting light intensity, exposure time, and illuminated area. For example, GelMA-based hydrogel properties depend on MA substitution, GelMA concentration, initiator concentration, and light exposure time [112,114], which can be conveniently adjusted for various applications in MTE [54,55]. However, the potential cellular cytotoxicity and genotoxicity inherent to the photo-crosslinking reaction should be focused on [115].

#### 3.1.2. Schiff Base Reaction

The Schiff base reaction is commonly observed in the formation of dynamic covalent imine bonds between aldehyde and amine groups [46]. This reaction is pH-sensitive, mild, and biocompatible [46]. Due to its reversible property, it enhances the stability of hydrogel networks and provides self-healing properties, making them suitable for the injection and delivery of living cells and drugs in situ [117,118]. Chen et al. developed an injectable hydrogel system based on the Schiff base reaction for meniscus repair [37]. They achieved this by employing a multi-aldehyde modified four-arm polyethylene glycol (4-arm PEG-CHO) that reacted with amino groups in glycol chitosan. Through this approach, they successfully delivered MSCs and transforming growth factor β (TGF-β) to repair meniscus defects, which was demonstrated in an in vivo rabbit model [37]. Similarly, Resmi et al. developed a self-crosslinking injectable hydrogel system based on the Schiff base reaction using alginate dialdehyde (ADA) and gelatin to promote the healing of longitudinal meniscal tears. In an in vitro culture model, the hydrogel demonstrated excellent integration with the host meniscal tissue.

#### 3.1.3. Crosslinking Agents

This is a convenient and simple way to form covalent bonds between hydrogel precursors by incorporating crosslinking agents. Glutaraldehyde, for instance, is a commonly used small-molecule crosslinking agent in hydrogel formation. Glutaraldehyde possesses two aldehyde groups, thereby enabling it to create imine bonds, bridging two amino groups within polymers (Figure 3c). Zhang et al. used this strategy to form gelatin-based meniscus implants for healing meniscus avascular defects [52]. However, the use of glutaraldehyde raises safety concerns due to its toxicity, even though it is a cheap and easy crosslinking method. In comparison, genipin, a natural crosslinker derived from plants, offers superior performance. The crosslinking mechanism between genipin and primary amine is also based on the Schiff base reaction, but genipin only exhibits 0.01% of the cytotoxicity of glutaraldehyde, thereby demonstrating higher biosafety [119]. Tarafder et al. successfully utilized genipin to crosslink FB to enhance the stability and mechanical properties of fibrin hydrogel [38]. This strategy effectively retards the degradation rate of the hydrogel without compromising its biocompatibility [38].

#### 3.1.4. Enzyme-Mediated Crosslinking

Enzymes are known as biological catalysts that are able to speed up biochemical reactions in living organisms. These reactions occur naturally under mild conditions that are compatible with biological systems. As a result, researchers have explored using various enzymes to promote the crosslinking of polymers and the formation of hydrogels. As shown in Figure 3d, tyrosinase-mediated crosslinking is a very gentle process that involves the oxidation of tyramine to produce active quinone groups, which then promptly form covalent bonds with each other [60]. Kim et al. used this highly cell-friendly method to crosslink TA-HA and form an injectable hydrogel for meniscus repair. Another exemplary case of enzyme-mediated crosslinking is the transversion from fibrinogen to fibrin triggered by thrombin. This enzymatic reaction has facilitated the use of fibrin pre-gel mixtures as cell carriers, wound-healing systems, and bio-adhesives [84]. As stated above, the utilization of fibrinogen and thrombin as a delivery method for cells and active additives has gained significant popularity in the field of MTE.

#### 3.1.5. N-Hydroxysuccinimide-Ester (NHS-Ester) Chemistry

The NHS-ester-mediated reaction is a highly efficient process that facilitates nucleophilic attack and enables the rapid formation of crosslinks with primary amines [46]. This reaction can occur under mild and biocompatible conditions, making it widely utilized in various bioconjugation applications such as peptide synthesis and protein labeling [120]. Furthermore, in the field of biomaterial research, it serves as a common crosslinking strategy for hydrogel formation [121]. A study by Simson et al. synthesized chondroitin sulfate-NHS, which bound living cells and bone marrow proteins [67]. As a result, injectable hydrogels were developed to possess the capability of adhering to meniscal tissues. These hydrogels not only provide mechanical stability to the tissue repair interface but also promote tissue regeneration at the site of injury [67].

#### 3.1.6. Radiation-Induced Crosslinking

Radiation-induced crosslinking is widely acknowledged as a method to create pure and sterile hydrogels [122]. As shown in Figure 3f, in the case of radiation-induced crosslinking, crosslinks are formed by the irradiation-induced generation of abundant radicals. During γ-radiation, water absorbs energy and produces hydroxyl radicals (·OH). These radicals activate polymers by extracting hydrogen, creating numerous free radicals, which then covalently crosslink, forming a hydrogel network [41]. The radiation crosslinking method offers several advantages. It eliminates the need for crosslinking agents and ensures the absence of any residual toxic substances in the hydrogel [123]. Moreover, the degree of crosslinking can also be easily controlled by changing the irradiation dose [123]. Li et al. recently used γ-irradiation to construct SF hydrogels. The hydrogels were then modified to bind bioactive peptides, which led to the recruitment of endogenous stem cells and effectively promoted meniscus repair [41].

### 3.2. Non-Covalent Crosslinking

Non-covalent crosslinking forms reversible bonds between various components. Ionic crosslinking (Figure 3g), electrostatic interactions (Figure 3h), hydrogen bonds (Figure 3i), and physical entanglement (Figure 3j) are examples of non-covalent mechanisms that have been extensively studied and utilized in the development of hydrogel systems for MTE research.

#### 3.2.1. Ionic Crosslinking

Ionic crosslinking (Figure 3g) between negatively charged functional groups and metal ions is one of the most common crosslinking strategies used for hydrogel formation [115,116]. For example, alginate can be crosslinked by numerous multivalent cations such as calcium ion (Ca^2+^), magnesium ion (Mg^2+^), aluminum ion (Al^3+^), and iron ion (Fe^3+^) to form hydrogels, in which carboxylic groups of adjacent polymers interact with these ions to generate so-called “egg box” structures [116,124]. Using this rapid and reversible strategy, the alginate-based hydrogel can form at room temperature and physiological pH, making it an attractive material for in situ injection to adaptably fill the complex meniscus tears [64]. In addition, the alginate/Ca^2+^ combination can also serve as an ideal cell or drug carrier for the infusion of 3DP scaffolds in creating composite meniscus scaffolds [62,63].

#### 3.2.2. Electrostatic Interaction

Electrostatic interaction occurs between polymer chains with opposite charges (Figure 3h). For instance, carboxyl groups in Alg can react with amino groups in gelatin or chitosan [51,125]. This reaction does not require any crosslinking agent, is mild, and is biocompatible. However, its bond strength is relatively limited, and the resulting hydrogel often necessitates additional crosslinking in MTE research to achieve higher crosslinking density. In the hydrogel studied by Resmi et al., some carboxyl groups in Alg could form electrostatic interaction with amino groups, whereas the others were oxidized to introduce aldehyde groups [51]. These aldehyde groups formed imine bonds with gelatin, enhancing the mechanical properties and stability of the hydrogel network [51].

#### 3.2.3. Hydrogen Bonds

Hydrogen bonds (H-bonds) form as the intermolecular interactions between the hydrogen atoms and electronegative atoms, such as oxygen (O) or nitrogen (N) atoms (Figure 3i) [126]. They play a critical role in the formation of three-dimensional structures for biomacromolecules such as proteins [127]. COL I and m-dECMs can self-assemble into injectable hydrogels as the result of H-bond formation between amino acid residues within peptide chains in COL I under physiological pH conditions, such as proline residues and hydroxyproline ones (Figure 3i) [73,128]. Without any crosslinker and harsh gelation conditions, COL I or m-dECMs can be easily prepared for cell delivery, thereby making them frequently used in MTE. Moreover, due to containing hydroxyl groups in their backbone, abundant H-bonds are formed in natural polysaccharides [126]. For instance, cellulose contains abundant H-bonds in the crystallites and provides exceptional mechanical properties for reinforcing hydrogel [126]. Therefore, H-bonds have commonly been employed as a reversible crosslinking mechanism for hydrogel formation, and multiple H-bonds can be utilized to enhance the mechanical strength of hydrogels.

#### 3.2.4. Physical Entanglement

Physical entanglement (Figure 3j), another common nonvalent interaction utilized in creating polymer networks for hydrogelation, begins with peptides evolving into nanofibers that subsequently entangle to form hydrogels with a nanofibrous network [79]. One example is the aforementioned peptide KI24RGDS, which features self-crosslinking ability, ease of preparation, and good injectability for repairing partial meniscus defects [79]. However, this crosslinking method is thought to be insufficient in constructing polymer networks for hydrogelation [129], necessitating additional crosslinking to solidify the hydrogel three-dimensional networks.

### 3.3. Pros and Cons

Crosslinking is a necessary and crucial process for hydrogel formation. Various covalent and non-covalent strategies have been extensively explored in the application of MTE. Covalent ones have stronger bond strength, thereby generating stable networks in hydrogel permanently [46,130]. However, complex chemical modification, expensive crosslinkers (e.g., genipin and enzymes), or cheap but toxic reagents (e.g., aldehydes) are required. In addition to dynamic covalent bonds, other covalent crosslinks in hydrogels are often permanent and rigid [130,131]. Nonetheless, a lot of hydrogels are too brittle to endure the high covalent-bonding strength, leading to hydrogel break and fracture when exposed to external force. In comparison, dynamic covalent crosslinking and non-covalent strategies are relatively weak in bond strength but possess reversible characteristics with versatile application situations, such as injectable hydrogels and shear-thinning hydrogels [51,64,68,132]. Moreover, they need fewer additional chemical crosslinkers or chemical modifications. Therefore, poisonous crosslinkers like aldehydes can be avoided and the biological properties of raw materials are less likely to be influenced. Nonetheless, reinforcement mechanisms should be introduced to improve mechanical performance in non-covalent crosslinked hydrogels.

## 4. Therapeutic Additives

Various therapeutic additives, including cells, growth factors, drugs, and other bioactive components, have frequently been encapsulated within different hydrogel systems to enhance their overall performance. These additives can work synergistically with hydrogel substrates to facilitate meniscus repair and regeneration by stimulating regenerative mechanisms. This stimulation promotes cell proliferation, migration, and differentiation and reduces inflammation, among other effects. A hydrogel system often incorporates various additives to achieve multiple biological functions, whereas different hydrogel designs may choose to deliver the same individual additive. Some examples of therapeutic additives loaded by different hydrogel application scenarios have been summarized in Table 2. It should be pointed out that the loading methods have a significant impact on the final performance generated by therapeutic additives, in addition to their properties. Therefore, in this section, we start our overview of widely utilized loading methods and will delve into the biological roles of the additives after that.

### 4.1. Loading Methods

**Figure 4:**
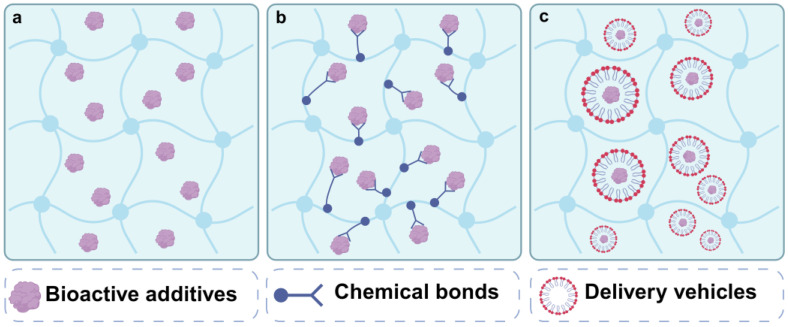
Schematic diagram of loading methods for therapeutic additives in hydrogels for MTE: (**a**) physical entrapment; (**b**) covalent binding; (**c**) incorporation of other vehicles. The figure was created with BioRender.com.

To achieve ideal biological performances, the controlled release of therapeutic additives loaded by hydrogels must be considered. The release process is influenced by numerous factors, ultimately impacting the subsequent bioactivities and functions of the additives. For instance, the additive release can be manipulated with the tuning parameters of the hydrogel carriers, such as pore size, backbone charge, hydrophilicity, or crosslink density, as these factors directly affect the diffusion rates and solubility of molecules dispersed within the hydrogel [135]. The interactions between hydrogels and additives, including covalent linkages or electrostatic attractions, can influence the additive release profiles. Additionally, the physiochemical properties of therapeutic additives impact their release and utilization. For example, hydrophobic bioactive agents have poor solubility in hydrophilic hydrogels, which retain large amounts of water and hinder their bioavailability. Therefore, when loading therapeutic additives into hydrogel systems, appropriate methods must be employed. Numerous biomaterials and strategies have been developed for therapeutic additive encapsulation. Herein, we focus on the main methods associated with hydrogel systems for MTE, including physical entrapment within hydrogels (Figure 4a), covalent binding to hydrogels (Figure 4b), and incorporating other vehicles to load additives (Figure 4c).

#### 4.1.1. Physical Entrapment

Physical entrapment is a simple method of loading cells and bioactive additives through direct encapsulation. Before gelation, the precursors can be mixed with cells and/or additives, which are then encapsulated into the solidified hydrogel via the curing process. Most pure protein-based hydrogels, such as collagen, gelatin, fibrinogen, and ECM hydrogel, can be degraded by the encapsulated cells or the surrounding cells after implantation in vivo, causing the release of additives. For example, FB hydrogel has good biocompatibility, but its rapid degradation rate leads to a burst release effect. It has been reported that 70% of the initially encapsulated additives are released into the surrounding environment within only one day [136]. As mentioned above, manipulation of the crosslink density can slow down the degradation rate of the hydrogels, such as increasing the concentrations of thrombin and fibrinogen [137] or introducing other crosslinking agents, such as genipin [38]. The tunability of hydrogels can also be enhanced through chemical modification. As an example, the crosslinking process of the aforementioned GelMA hydrogel can be adjusted by controlling the duration of light exposure [54]. Following UV irradiation for just 30 s, the researchers found that TGF-β3 could be released sustainably from the GelMA hydrogel for three weeks in vitro. In addition, non-covalent interaction between hydrogel components and additives is used to mitigate the release rate. For instance, the incorporation of heparin into hydrogels is a facile strategy that takes advantage of the well-known electrostatic attraction between heparin and protein [38]. Overall, the crosslinked networks in hydrogels effectively prevent the penetration of various proteins, preserving additives from premature degradation by inwardly diffusing enzymes and enhancing the long-term retention rate of additives at the target site [36]. However, there is currently a lack of precise control over the release rate of additives through solely physical entrapment [138].

#### 4.1.2. Covalent Binding

The covalent binding of bioactive additives to hydrogels necessitates the modification of polymer matrices to incorporate reactive groups such as NHS, aldehyde, and maleimide that are capable of forming covalent bonds with functional groups present in the additives, like amino or thiol groups [37,39,41,67]. For instance, Li et al. employed sulfosuccinimidyl 4-N-maleimidomethyl to functionalize SF hydrogel infused within PCL scaffolds [41]. The maleimide groups in the hydrogel rapidly react with thiol groups in a peptide, named L7, which exhibits a high affinity for binding with MSCs. Analogously, the swift interaction between the NHS ester and amino group not only facilitates crosslinking for hydrogel formation but also enables binding with additives, including growth factors and other proteins, for controlled release [39,67]. Xu et al. introduced TGF-β3 and CTGF into a PEG-NHS solution, which was subsequently mixed with PEG-NH_2_ and infused into a 3D-printed PCL scaffold to create a composite meniscus-like construct [39]. They observed that the loaded growth factors could be released over 21 days. Generally, the release kinetics of additives that are covalently bound within hydrogel backbones offer long-term control over their availability. The sustained release process is often mediated by hydrogel matrix degradation rather than diffusion [137], which prevents sudden release, rapid degradation, and fast clearance of the additives, ultimately enhancing therapeutic effectiveness. [36,138]. However, covalent binding may obstruct active sites on the additives, such as growth factors or other proteins, posing the risk of altering their molecular conformations and diminishing their bioactivity [138]. In comparison, dynamic covalent linkages such as imine bonds, mediated by the Schiff base reaction, may offer a gentler loading process for growth factors and enable controlled release. The aforementioned injectable hydrogels, composed of chitosan and 4-arm PEG-CHO, have been reported to achieve stable sustained release of TGF-β1 for 14 days, with no significant initial burst release [37].

#### 4.1.3. Incorporation of Other Delivery Vehicles

The third approach to integrating bioactive additives is the incorporation of alternative delivery vehicles—micelles [52], nanoliposomes (NLPs) [24], microspheres (MPs) [58,71,134], and nanoparticles (NPs) [71,134]—within hydrogels. These vehicles address a fundamental challenge: the inherent hydrophobicity and insolubility of certain drugs or agents in aqueous environments, which limits their bioavailability. Moreover, embedding bioactive additives within these vehicles confers exceptional control over release kinetics. This precision allows for tailored, long-term sustained or sequential release patterns tailored to specific applications and ultimately enhancing delivery efficiency in comparison to conventional methods. Micelles, notable for their amphiphilic nature, effectively solubilize hydrophobic molecules. In a recent study, L-lactic acid oligomer–gelatin micelles were utilized for the sustained release of the hydrophobic drug simvastatin, demonstrating therapeutic efficacy in meniscus healing [52]. NLPs, composed of a phospholipid bilayer, uniquely accommodate both hydrophilic and hydrophobic molecules. A fascinating development by Liu et al. introduced a sustained drug delivery system leveraging NLPs [24]. This system, composed of phosphatidylcholines, not only facilitates drug loading but also reduces friction between cartilage surfaces, offering a dual benefit of drug delivery and knee joint protection from wear. In the following sections, we will explore the details of the system’s design and its functions. Li et al. combined m-dECM hydrogel with poly (lactic-co-glycolic acid) (PLGA) MPs loaded with kartogenin (KGN), a hydrophobic molecule known for its chondrogenic effect [71]. It was observed that KGN exhibits an initial burst release from the MPs within the first 3 days, followed by a gradual release that becomes increasingly sustained over 28 days, resulting in a cumulative release rate of 78%. Furthermore, the same team utilized PLGA NPs to encapsulate CTGF and PLGA MPs to carry TGF-β3. This approach achieved sequential release, as the NPs degraded more rapidly, leading to a prompt release of CTGF. This technique can effectively introduce heterogeneous meniscus tissue types [134]. Although delivery vehicles possess many advantages, fabricating these delivery vehicles requires additional and complex steps that are relatively time-consuming and costly.

### 4.2. Cells

**Figure 5:**
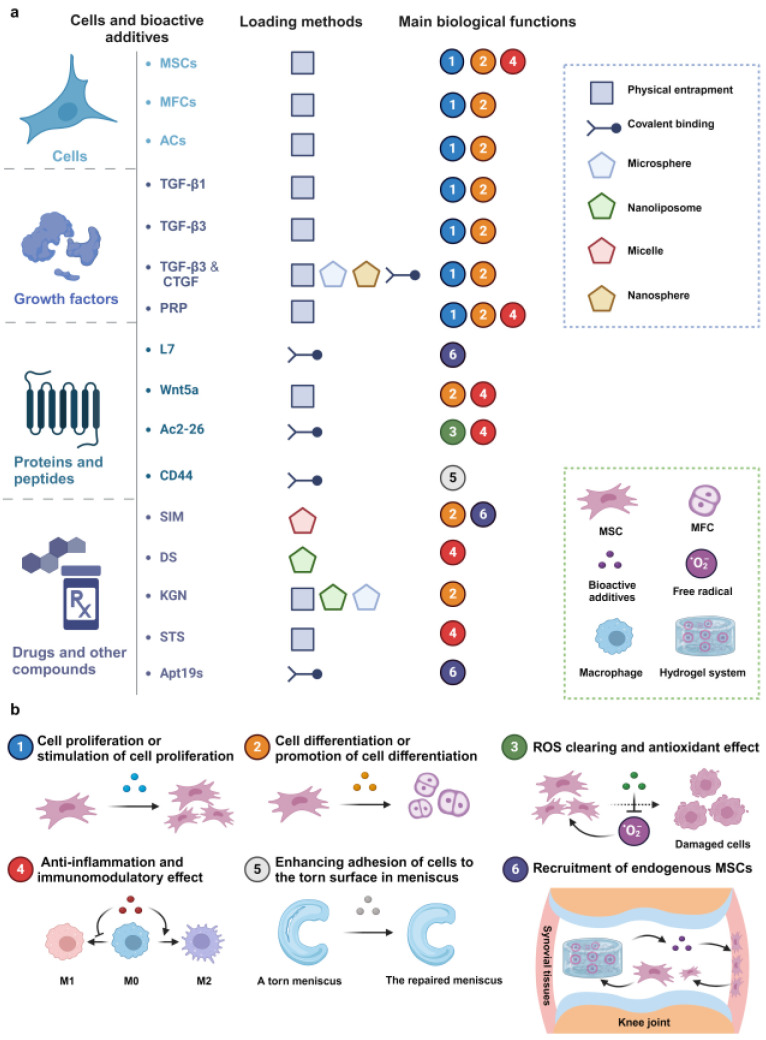
The main biological functions of cells and bioactive additives loaded by hydrogels in MTE: (**a**) classification of cells and bioactive additives, MSCs: mesenchymal stem cells, MFCs: meniscus fibrochondrocytes, ACs: articular chondrocytes, TGF-β1/3: transforming growth factor-β1/3, CTGF: connective tissue growth factor, PRP: platelet-rich plasma, L7: LTHPRWP (L7) peptide, Wnt5a: wingless-type MMTV integration site family member 5a, Ac2-26: annexin-1 mimetic peptide, CD44: cluster of differentiation 44, SIM: simvastatin, DS: diclofenac sodium, KGN: kartogenin, STS: sodium tanshinone IIA sulfonate, Apt19s: a kind of MSC-specific aptamer; (**b**) biological functions of cells and bioactive additives, ROS: reactive oxygen species, M0: macrophage, M1: pro-inflammatory macrophage, M2: anti-inflammatory macrophage. The figure was created with BioRender.com.

Cell-based therapy is among the strategies most based and heated in tissue engineering and regenerative medicine [46]. MSCs, meniscus fibrochondrocytes (MFCs), and articular chondrocytes (ACs) are the most used cells. The integration of MSCs, MFCs, and ACs with hydrogels is essential for effective meniscus repair and regeneration, as shown in Figure 5a,b.

#### 4.2.1. Mesenchymal Stem Cells (MSCs)

As a non-hematopoietic subpopulation of cells, MSCs have multi-lineage potential to differentiate into various tissues of mesodermal origin [139]. MSCs from various tissues, such as bone marrow-derived MSCs (BMSCs) [23], adipose-derived MSCs (ASCs) [54], tonsil-derived MSCs (TMSCs) [47], and so on, have been extensively explored and delivered through hydrogel for meniscus repair and regeneration. In terms of their function, MSCs can differentiate into meniscus cells under specific conditions. Not only have MSCs been proven to promote the phenotype of meniscal cells but they can also prevent meniscus cell hypertrophy in co-culture models [140,141]. In addition, animal models of transplantation MSCs confirmed that these cells promote donor-specific tolerance through the generation of regulatory T cells and antigen-presenting cells, thereby supporting their potential immune tolerance-inducing effects [142]. Moreover, MSCs can mitigate local inflammation by inducing macrophage polarization to anti-inflammatory phenotypes and increase the secretion of interleukin-10 (IL-10) [143]. Interestingly, a team reported that MSCs cultured in a 3D hydrogel system exhibited superior inflammatory modulation and regenerative potential compared to those expanded on a 2D substrate due to the increased prostaglandin E2 (PGE2) secretion by MSCs cultured in a 3D environment [144]. These findings suggest that hydrogel systems offer advantages for MSC delivery and that the ex vivo expansion of MSCs for MTE should be optimized by transitioning from traditional 2D culture dishes to 3D culture systems.

#### 4.2.2. Meniscus Fibrochondrocytes (MFCs) and Articular Chondrocytes (ACs)

MFCs and ACs are terminally differentiated cells that possess specific phenotypes. These cells are extracted and harvested from native meniscus and articular cartilage tissues. Therefore, they serve as a direct supplement in healing meniscus injuries by replenishing the cell pool and promoting tissue homeostasis and regeneration. To obtain sufficient autologous cells, ex vivo cell amplification is necessary. However, during in vitro cell culture, there is an increasing tendency for these cells to dedifferentiate and lose their phenotype [145], thus limiting their application for use in MTE.

### 4.3. Growth Factors

The delivery of growth factors (GFs) alongside cells has been a common approach to enhancing meniscus regeneration, as these GFs play crucial roles in regulating cell proliferation, migration, and differentiation (Figure 5b). Furthermore, given the zone-specific or heterogeneous tissue phenotypes observed in native meniscus tissues, the spatial and sequential release of multiple GFs has been demonstrated to effectively induce the heterogeneous tissue phenotypes of the meniscus in both in vitro and in vivo models [39,134]. It is worth mentioning that numerous GFs are commonly involved in the fibrochondrogenic differentiation of MSCs and the maintenance of meniscus cell phenotype. For this discussion, we will concentrate on the most frequently utilized GFs in hydrogel systems.

#### 4.3.1. Transforming Growth Factor-β (TGF-β)

TGF-β, as one of the main members of the TGF-β superfamily, plays critical roles in cell proliferation, embryogenesis, and adult tissue homeostasis [146]. It has been studied for tissue regeneration for several decades [146]. The isoforms of TGF-β, including TGF-β1, TGF-β2, and TGF-β3, are potent agonists of chondrocyte differentiation and govern cartilage physiology and pathology [146]. Because articular cartilages and the inner tissues of the meniscus share similar phenotypes, most research groups have adopted TGF-β members, mainly referring to TGF-β1 and TGF-β3, to induce meniscus-like tissues in MTE, as mentioned above [37,54]. Previous studies have revealed that TGF-β1 promotes chondrogenic differentiation of BMSCs in part through the indirect downregulation of N-cadherin, which leads to the relaxation of cytoskeletal tension, reduced Ras homolog gene family member A (RhoA) and its downstream effector Rho-associated protein kinase (ROCK) (RhoA/ROCK) signaling, and upregulation of chondrogenic signaling and gene expression, such as sex-determining region Y-related high-mobility group-box gene 9 (Sox-9) [147,148]. TGF-β3 shares a similar signal transduction pathway but is considered to possess greater chondrogenic effects than TGF-β1 [146].

#### 4.3.2. Connective Tissue Growth Factor (CTGF)

CTGF, also known as cellular communication network 2 (CCN2), is involved in many biological functions, such as cell proliferation, migration, adhesion, wound healing, and angiogenesis [149,150]. CTGF binds to TGF-β through its von Willebrand factor C (VWC) domain and thrombospondin type 1 repeat (TSR) domain. During chondrogenesis, the interaction between CTGF and TGF-β1/TGF-β3 promotes chondrocyte differentiation and endochondral ossification [151]. In addition, CTGF acts synergistically with TGF-β1 to promote matrix protein deposition and mediate fibrogenesis [152]. Tissues in the outer region of the meniscus exhibit a highly fibrotic phenotype characterized by abundant collagen bundles. Therefore, CTGF has been frequently used in MTE to induce fibrotic tissue formation in this area. CTGF and TGF-β have emerged as a classic combination for chemically inducing MSC differentiation with the aim of generating a desired heterogeneous phenotype [39].

#### 4.3.3. Platelet-Rich Plasma (PRP)

PRP is a concentrated platelet product derived from autologous blood, containing growth factors and cytokines like TGF-β and IL-1RA [153,154]. PRP has shown the capacity to inhibit inflammation and improve cartilage tissue regeneration by promoting the synthesis of cartilage matrix, increasing cell growth and migration, and inducing phenotype changes [133,153]. In the field of meniscus tissue engineering (MTE), PRP has been incorporated into various hydrogel systems and has demonstrated high potential for meniscus repair [132,133,155,156,157,158,159]. It is worth mentioning that PRP contains fibrinogen, which can be activated by thrombin and Ca^2+^ to form a hydrogel. Liu et al. have taken advantage of this characteristic to load BMSCs and KGN into PRP hydrogel [132]. Furthermore, when meniscus wounds are treated with BMSC-containing KGN-PRP hydrogel in vivo, they produce more cartilage-like tissue [132]. Hagmeijer et al. used PRP to coat Collagen Meniscus Implants (CMI^®^), which increased the migration of meniscus cells and MSCs into the implant [156]. These findings offer promising results for the use of PRP in meniscus regeneration. However, conflicting outcomes have also been reported. Lee et al. found that PRP treatment increased the presence of catabolic molecules associated with IL-1α-induced inflammation [160]. Furthermore, PRP treatment did not promote meniscus cartilage formation but rather accelerated fibrosis in an in vivo meniscus injury model [160]. In light of these conflicting results, caution should be exercised when using PRP in MTE, and further evaluation is needed to fully understand its role in meniscus regeneration.

### 4.4. Proteins and Peptides

#### 4.4.1. LTHPRWP Peptide (L7)

L7, identified by a research group using phage display technology, is a seven amino acid peptide sequence with a high specific affinity to MSCs [161,162]. Using this discovery, L7 has been conjugated with PCL to recruit endogenous homing and promote tissue regeneration [161,162]. As discussed earlier, Li et al. used a covalent-binding strategy to conjugate L7 with SF and create a cell-free scaffold with PCL for meniscus regeneration. This demonstrates the potential of the cell-free strategy in stimulating endogenous cell in-growth while avoiding the challenges and risks associated with using exogenous cells, such as ethical concerns, expensive cell amplification, and more [41].

#### 4.4.2. Wingless-Type MMTV Integration Site Family Member 5a (Wnt5a)

Wnt5a is a crucial non-canonical Wnt signaling ligand that plays a vital role in the development of musculoskeletal tissues, including cartilage [163]. Farrera-Hernández et al. showed that Wnt5a activated calcium release in the undifferentiated region during digit development [164]. Furthermore, they confirmed that the non-canonical Wnt5a-Ca^2+^-calcineurin (CaN) nuclear factor of activated T-cell (NFAT) signaling pathway played a crucial role during embryonic digit development in vivo, enhancing chondrogenic signaling competence [164]. In MTE research, Qi et al. loaded Wnt5a into PRP gels and demonstrated that implantation of Wnt5a/PRP gels promoted significant meniscal defect regeneration in a rabbit model [133]. Moreover, they observed that PRP and Wn5a exhibited synergistic anti-inflammatory effects by regulating the nuclear factor-κB (NF-κB) signaling pathway.

#### 4.4.3. Annexin-1 Mimetic Peptide (Ac2-26)

Ac2-26, a peptide derived from the N-terminus of annexin A1, has been identified as a strong inflammation-resolving mediator that can be combined with biomaterials to treat inflammatory diseases [165,166]. Mechanistically, Ac2-26 binds to formyl peptide receptor (FPR) 2, a seven-transmembrane G protein-coupled receptor expressed on the surface of macrophages, to activate the FPR signaling pathway and its anti-inflammatory effects [165,167]. Currently, the impact of the inflammatory microenvironment on meniscus regeneration has been emphasized, particularly in the context of meniscal injuries or invasive surgery for meniscus scaffold transplantation, where damage-associated molecular patterns (DAMPs) are involved. To mitigate the adverse effects of heightened inflammation, Xu et al. conjugated Ac2-26 with PEG-NHS-based hydrogels and infused them into 3DP PCL scaffolds to modulate the inflammatory microenvironment during meniscus regeneration [39]. The report indicates that the integration of Ac2-26 into meniscus scaffolds not only reduces the levels of reactive oxygen species (ROS) but also alleviates inflammation by shifting macrophage phenotype from pro-inflammatory M1 to anti-inflammatory M2 in vitro [39]. However, the benefits of Ac2-26 in promoting meniscus regeneration should be further evaluated, as the authors have provided no further in vivo evaluations or evidence.

#### 4.4.4. Cluster of Differentiation 44 (CD44)

CD44, a type of cell-adhesion molecule, and its associated partner proteins govern changes in the ECM that affect cell growth, survival, and differentiation [168]. As previously mentioned, the interaction between CD44 and HA is crucial in mediating meniscus regeneration. However, a recent study reported that the pre-deposition of HA on the torn meniscus surface leads to lubricin deposition, which can have adverse effects on meniscus healing [38]. This is because lubricin has an inherent property that prevents cell and protein adhesion [169]. To address this issue, researchers have devised a strategy using an FB-based hydrogel conjugate with CD44, possessing a high affinity for HA and lubricin [38], which, in return heals meniscus tears because CD44 reduces the negative impact from the deposition of HA/lubricin onto the surface of the torn meniscus. To further enhance the affinity between the hydrogel and CD44 as well as HA/lubricin, they utilized heparin to modify this hydrogel system [38]. By incorporating TGF-β3-loaded PLGA MPs and CTGF into this hydrogel system, they found that the hybrid hydrogel-tethering lubricin holds great potential for enhancing the healing of avascular meniscus tears [38].

### 4.5. Drugs and Other Compounds

#### 4.5.1. Simvastatin (SIM)

Simvastatin as the competitive inhibitor of 3-hydroxy-3-methylglutaryl coenzyme A reductase has been widely used for hyperlipidemia in clinics. However, simvastatin has recently been extensively studied for its diverse biological functions beyond cholesterol reduction, including chondrogenic effects [170]. Zhang et al. demonstrated that this drug can stimulate the healing of avascular menisci in a rabbit model [52]. To achieve sustained drug release, they encapsulated the drug into L-lactic acid oligomer–gelatin micelles, which were then integrated into gelatin hydrogels crosslinked by glutaraldehyde. The simvastatin-loaded hydrogels exhibited enhanced meniscus tissue regeneration after implantation into defects at 12 weeks. Immunohistochemical analysis of the regenerated tissues revealed stronger positive staining for COL I, COL II, bone morphogenetic protein 2 (BMP-2), and BMP-7 in the simvastatin group compared to the control group. It is speculated that the simvastatin-induced increases in BMP-2 and BMP-7 mediated meniscus regeneration. In addition, simvastatin promotes the migration and homing of MSCs [171], providing another potential mechanism for enhancing meniscus tissue regeneration [52].

#### 4.5.2. Diclofenac Sodium (DS)

DS is a type of nonsteroidal anti-inflammatory drug (NSAID) commonly prescribed for relieving pain and reducing inflammation associated with arthritic joint diseases such as osteoarthritis (OA) [172]. It exerts its anti-inflammatory effect by inhibiting cyclooxygenase-2 (COX-2) expression in inflammation-induced macrophage cells [173]. Recently, it has been incorporated into tissue-engineered scaffolds, exhibiting its capacity to suppress local excessive inflammation [174]. Liu et al. recently used nanoliposomes to load DS and KGN, which were then incorporated into a gelatin-based hydrogel for meniscus repair and OA prevention [24]. DS, being a hydrophilic drug, rapidly releases from the carriers within 48 h, effectively reducing acute inflammation. However, KGN, due to its unique hydrophobic nature, exhibits slow-release behavior and serves as a long-term chondrogenic inducer. These additives work synergistically, enhancing the therapeutic effects.

#### 4.5.3. Kartogenin (KGN)

In 2012, Kristen Johnson and his colleagues first reported the discovery of a structurally simple compound called KGN [175]. This compound has been found to possess a potent ability to stimulate the differentiation of MSCs into chondrocytes. Remarkably, this effect is observed even at remarkably low concentrations, as little as 100 nM. The chondrogenic mechanism of KGN is that it binds to filamin A, interfering with its interaction with the transcription factor core-binding factor β subunit (CBFβ), and regulates the CBFβ-RUNX1 transcriptional programs to induce chondrogenesis [175]. However, KGN has a naturally hydrophobic characteristic, thereby requiring chemical binding or vehicles to load for application in MTE, such as MPs [71] and nanoliposomes, as mentioned before [24].

#### 4.5.4. Sodium Tanshinone IIA Sulfonate (STS)

STS is a natural compound extracted from the traditional Chinese herb Salvia miltior-rhiza Bunge, also known as Danshen [176]. Not only does STS possess a strong anti-inflammatory effect due to its quinone structure but it has also demonstrated its capability of suppressing chondrocyte dedifferentiation [62,177]. Recently, Li et al. loaded STS into an Alg/m-dECM hybrid hydrogel and used this hydrogel to infuse a 3DP PCL backbone and generate a composite meniscus scaffold [62]. They confirmed that STS could induce macrophages to transform from the pro-inflammatory phenotype (M1) to the anti-inflammatory phenotype (M2). Furthermore, STS prevented ECM degradation in MFCs by reducing interleukin (IL)-1β-induced inflammation, oxidative stress, and apoptosis, which was achieved through the inhibition of the IL-1 receptor-associated kinase 4 (IRAK4)/tumor necrosis factor receptor (TNFR)-associated factor 6 (TRAF6)/NF-κB signaling pathway [62]. The scaffolds seeded by MFCs also exhibited good meniscus regeneration and chondroprotective effects in rabbits.

#### 4.5.5. Aptamer-Apt19S (Apt19S)

Aptamers are short nucleic acid or peptide sequences that can specifically recognize a wide range of targets, from small molecules to entire cells [178]. The small molecular components, which exhibit high specificity and affinity for target ligands, have the advantage of lacking immunogenic or toxic side effects [134]. They hold great potential in selectively recruiting MSCs to sites of tissue defects, initiating and promoting endogenous mechanisms for tissue repair and regeneration [134,178]. Recently, the DNA aptamer Apt19s was developed and conjugated with m-dECMs through covalent binding, aided by 1-ethyl-3-(3-dimethylaminopropyl) carbodiimide hydrochloride (EDC) and NHS [134]. These modified ECMs were then integrated into GelMA containing TGF-β3-loaded MPs and CTGF-loaded NPs. The composite meniscus scaffolds were formed by infusing this mixture into 3DP PCL scaffolds and curing. It has been shown that these cell-free scaffolds can mobilize endogenous MSCs both in vitro and in vivo. Furthermore, they promote fibrocartilaginous differentiation and meniscus regeneration through the synergistic effect of Apt19s and the sequential release of CTGF and TGF-β3 in rabbit models.

### 4.6. Pros and Cons

Physical entrapment is a widely used and convenient strategy to anchor cells and additives at the target area. However, its main limitation is the lack of precise control over the release mechanism. The covalent binding approach provides stable covalent linkages between additives and the polymer network, ensuring the retention of the additives until the polymers degrade. Nevertheless, careful attention should be paid to avoid altering the bioactivity of the additives. The use of micro-/nanoscale carriers offers precise control over the release rate and protection for the additives. Expensive and time-consuming manufacturing protocols can be optimized through technical advancements. Overall, the choice of loading method depends on the specific requirements of the application and the desired release profile of the additives.

Regarding using cells and additives, there are some advantages and disadvantages. The delivery of therapeutic cells, particularly MSCs embedded in hydrogels, effectively promotes meniscus tissue repair and regeneration through cell proliferation and differentiation and the modulation of inflammatory and immune responses. However, several challenges persist in cell harvesting and application, such as high manufacturing costs and safety concerns over the potential tumorigenicity [179]. GFs have shown promising potential in clinical trials, but only a relatively small subset has achieved commercial success due to safety and cost-effectiveness concerns [180]. PRP serves as a natural reservoir for GFs, yet further evaluation is required for the aforementioned inconsistent and conflicting experimental results regarding meniscus repair. Other additives offer more targeted approaches, such as KGN for chondrogenic differentiation and DS for anti-inflammation. By targeting these specific processes, these additives have the potential to address specific needs in meniscus tissue repair and regeneration, though their long-term effects and safety remain uncertain. In summary, the use of cells and additives is often limited by their inability to address the broader range of biological processes involved in meniscus repair and regeneration, in addition to side effects. Therefore, further research is essential to gain a deeper understanding of the mechanisms of tissue repair and regeneration, which will not only aid in addressing current challenges but also inform the development of more effective and safer therapeutic strategies in the future.

## 5. Key Issues in the Design of Hydrogel Systems for Application in MTE

When designing a hydrogel for use in MTE, biocompatibility, biodegradability, and bioactivity are of the utmost importance. These properties are determined by the hydrogel components, crosslinks, and incorporated bioactive additives, which have been extensively discussed in previous sections. However, to effectively mimic the functions of the native meniscus in a real environment, several additional properties and key considerations must be taken into account, including shear-thinning properties, toughness, bio-adhesive properties, lubricating characteristics, and heterogeneity. Given that hydrogel has three primary applications in MTE—injectable hydrogels, hydrogel implants, and hydrogel-infused scaffolds, as illustrated in Figure 1—we will explore these crucial aspects through specific examples (presented in Table 2) in this section.

### 5.1. Shear-Thinning Property and Toughness

The property of shear-thinning makes a hydrogel’s viscosity decrease as the shear rate rises. During injection under shear stress, they behave like low-viscosity fluids but quickly revert to their solid-like state once the shear stress is removed [36]. Therefore, shear-thinning hydrogels exhibit an excellent balance between injectability and mechanical stability. When designing hydrogels, shear-thinning is a crucial factor that needs to be taken into consideration. Shear-thinning behavior is often caused by reversible crosslinks, such as dynamic covalent interactions (e.g., imine bonds) and non-covalent interactions (e.g., Ca^2+^ crosslinking; H-bonds). These crosslinking strategies and corresponding hydrogel component combinations such as PEG-CHO/chitosan [37], ADA/gelatin [51], alginate/Ca^2+^ [64], or m-dECMs [23,69,70] have been discussed in previous sections. However, these reversible hydrogel systems exhibit inferior toughness, making it challenging to maintain their structure under load-bearing conditions. To enhance hydrogel toughness, toughening mechanisms should be employed. For instance, semi-interpenetrating network hydrogels, such as the FB/PEO hydrogel [56] and the F127DA/nanocellulose hydrogel [55], have been utilized. Although injectable hydrogels and hydrogel implants are particularly well suited for treating partial meniscus defects, offering the potential to replace partial meniscectomy in clinical settings, pure hydrogels are typically too soft to effectively replace a fully damaged meniscus for patients with irreparable meniscus issues. Therefore, hydrogel-infused composite scaffolds remain necessary, as composite scaffolds combine the beneficial delivery properties and ECM mimicry of hydrogels with the structural robustness provided by strong biomaterials like PCL [39,40,41,43,62,63].

### 5.2. Bio-Adhesive Property

The bio-adhesive property of a hydrogel significantly impacts its performance. An exceptional tissue adhesion allows for better retention at the target site, ensuring the release of therapeutic agents at sufficient concentrations [36]. Additionally, the adhesive hydrogel plays a crucial role in tissue healing, as it closes torn interfaces more tightly compared to conventional hydrogels. This is particularly relevant when designing hydrogel systems used in MTE for meniscus tear healing. Given that the meniscus is surrounded by synovial fluid and experiences high stress, conventional hydrogels struggle to provide sufficient adhesion in this moist and loaded environment. Therefore, introducing an adhesive mechanism is necessary. For example, Simson et al. developed a meniscal adhesive hydrogel using NHS ester-modified CS. The NHS ester rapidly binds to amino groups on tissues and cells, enhancing meniscus repair and fusion [67]. Another common approach to improving hydrogel adhesion is introducing phenol groups, such as the example reported by Kim et al. [60]. Monophenol-based tyrosine residues can be oxidized by tyrosinase into reactive quinone groups that form covalent bonds with amino and thiol groups in native tissues, promoting adhesion. Furthermore, reducing the deposition of highly hydrophilic products on torn meniscus interfaces offers a novel approach to facilitating cell and protein adhesion by targeted delivery of CD44 that specifically binds to HA and lubricin [38].

### 5.3. Lubricating Property

A healthy meniscus is essential for providing a smooth interface and superior lubrication function, which plays a critical role in reducing friction as it articulates with the joint cartilage. Unfortunately, when using highly rigid tissue-engineered meniscus scaffolds such as PCL, this aspect is frequently overlooked, leading to severe cartilage wear [31]. Li et al. discovered that infusing PCL scaffolds with SF hydrogels resulted in a significant reduction in interface friction between the implant and the corresponding femoral condyle and tibial plateau [41]. This finding highlights another advantage of hydrogel systems concerning lubrication. As mentioned, Liu and colleagues developed a smart gelatin hydrogel system that possesses injectability, self-lubricating properties, and friction-responsive properties [24]. They used phosphatidylcholines to construct nanoliposomes that are designed to encapsulate and release drugs in a controlled manner. When exposed to friction within the knee joint, the drugs are released from the nanoliposomes in a sustained manner. Furthermore, the remaining nanoliposomes contribute to lubrication and minimize abrasion on the articular cartilage surface [24].

### 5.4. Heterogeneity

Meniscus tissues are known for their unique heterogeneous characteristics, making restoration of such complexity a pivotal goal in the field of MTE. Within this context, the application of hydrogel has emerged as a promising strategy. For example, Bahcecioglu et al. adopted a simple strategy of infusing different MFC-laden hydrogels into a pre-fabricated 3DP PCL scaffold and inducing the encapsulated MFCs to present zonally specific phenotypes [43]. They mixed MFCs with GelMA and Ag, which were infused and then anchored to the inner region of PCL scaffolds after photo-crosslinking. In contrast, only MFCs and GelMA were impregnated into the outer region of the scaffold. They observed that MFCs delivered by GelMA alone spread more evidently and produced more collagen in the outer region. On the other hand, the inner region exhibited lower cell adhesion, a round cell morphology, and higher levels of GAGs. This indicates that zone-specific hydrogel compositions can induce heterogeneous cellular phenotypes. Another approach to creating heterogeneity is through the use of sequential and zone-specific release techniques. For instance, Li et al. loaded TGF-β3 into PLGA MPs and loaded CTGF into PLGA NPs, which were then incorporated into GelMA/m-dECMs and infused into a 3DP meniscus-like PCL scaffold [134]. CTGF loaded in PLGA NPs released rapidly, inducing profibrogenic differentiation. In contrast, TGF-β3 in PLGA MPs remodeled the fibrous matrix into a fibrocartilaginous one through a long-term and slower release. Other researchers have used the same strategy and observed similar results [58,59]. Xu et al. created a customized mold to impregnate PEG hydrogels containing MSCs and TGF-β3 into the inner zone of a 3DP meniscus-like scaffold [39]. Meanwhile, hydrogels, MSCs, and CTGF were infused into the outer zone. The spatially specific release of CTGF and TGF-β3 within the same scaffold synergistically contributed to the heterogeneous differentiation of MSCs [39].

## 6. Conclusions

Hydrogels hold great promise in the field of meniscus tissue engineering due to their ECM-mimicking properties that are beneficial for cells, their ease of preparation and ability to load bioactive additives, and their versatility for clinical applications. This review summarizes a range of natural and synthetic biomaterials that can be crosslinked via covalent and/or non-covalent crosslinking to create hydrogels. These hydrogels, and drug vehicle-incorporated hydrogel systems in particular, are capable of encapsulating cells, growth factors, proteins, drugs, and other therapeutic agents. They play key biological processes such as cell proliferation, differentiation, antioxidation, and anti-inflammation while also recruiting endogenous stem cells to facilitate meniscus regeneration. In addition, the systems offer a broad range of applications in the field of MTE, including injectable hydrogels, hydrogel implants, and hydrogel-infused scaffolds. Although significant progress has been made in pre-clinical research, there are still challenges that need to be addressed.

Currently, the state-of-the-art in hydrogel development falls short of achieving true meniscus replacement. To move forward, future efforts in developing hydrogel systems for MTE use must focus on improving their toughness through developing high-strength hydrogels and composite meniscus scaffolds by combining hydrogels with strong 3D-printed scaffolds. Furthermore, to enhance the performance of hydrogels in healing torn meniscus interfaces, bio-adhesive mechanisms should be considered, such as incorporating rapid reactive groups, NHS ester, phenol groups, and more. To provide lubricating properties and prevent cartilage wear, the incorporation of biomaterials with lubrication such as phosphatidylcholines into hydrogels can be employed. The combined application of delivery vehicles within hydrogels can achieve the spatially and temporally specific release of growth factors (GFs), thereby restoring the meniscus’s heterogeneous phenotype, which is a core objective in MTE. However, tissue repair and regeneration involve complex dynamic signaling pathways within a microenvironment. The current hydrogels and delivery vehicles used to deliver GFs and other bioactive additives cannot fully recapitulate this dynamic process. Therefore, smart/stimuli-responsive hydrogel systems, such as programmable hydrogels with active drug delivery systems, are emerging as a promising future trend. To achieve these advancements, close collaboration between clinical medicine, developmental biology, chemistry and materials science, engineering, and computer science is essential. This multidisciplinary approach will enable the development of more effective hydrogels for meniscus tissue engineering and regeneration, paving the way for improved patient outcomes in the future.

## Figures and Tables

**Table 2:** Therapeutic additives loaded in hydrogels for various applications in MTE.

Hydrogels (Backbones)	Crosslinking Strategies	Cells	Additives (@Vehicles)	Models	Applications	References
Gelatin	Transglutaminase	None	KGN/DS@ NLPs	Cells*; rats	Injectable hydrogels	[24]
Gelatin; ADA	Schiff base reaction;	MFCs	None	Cells	Injectable hydrogels	[51]
Gelatin; TA-HA;	Tyrosinase	MFCs	None	Cells; in vitro explants^#^	Injectable hydrogels	[60]
FB; PEO	Thrombin; H-bonds	None	None	Rabbits	Injectable hydrogels	[56]
Alg	Ca^2+^ Crosslinking	None	None	Rabbits	Injectable hydrogels	[64]
GelMA; CNF; F127DA	Photo-crosslinking; H-bonds	None	None	Cells*	Injectable hydrogels	[54]
CS-NHS	NHS-ester chemistry	MFCs	BM	Cells; rats	Injectable hydrogels	[67]
Chitosan; β-GP	Electrostatic interaction	MSCs	None	Cells; nude mice	Injectable hydrogels	[68]
m-dECMs	H-bonds	MSCs; ACs	None	Cells; rats	Injectable hydrogels	[23,69,70]
4-arm PEG-CHO; Chi	Schiff base reaction	MSCs	TGF-β1	Cells; rabbits	Injectable hydrogels	[37]
Gelatin	Glutaraldehyde	None	SIM@Micelles	Cells*; rabbits	Hydrogel implants	[52]
FB	Thrombin	None	CTGF; TGF-β3@MPs	Cells*; in vitro explants^#^	Hydrogel implants	[58]
FB	Thrombin; genipin	None	CD44; TGF-β3@MPs; CTGF	Cells*; in vitro explants^#^	Hydrogel implants	[38]
PRP	Thrombin	MSCs	KGN	Cell; rabbits	Hydrogel implants	[132]
PRP	Thrombin/Ca^2+^	None	Wnt5a	Cells*; rabbits	Hydrogel implants	[133]
Alg; m-dECMs (3DP-PCL)	Ca^2+^ Crosslinking	MFCs	None	Cells; rats; rabbits	Hydrogel-infused scaffolds	[63]
SF (3DP-PCL)	Radiation-induced crosslinking	None	L7 peptide	Cells*; rats; rabbits	Hydrogel-infused scaffolds	[41]
PEG-NHS; PEG-NH_2_ (3DP-PCL)	NHS-ester chemistry	MSCs	Ac2-26; TGF-β3; CTGF	Cells	Hydrogel-infused scaffolds	[39]
m-dECMs (3DP-PCL)	H-bonds	None	KGN@MPs	Cells*; rats; rabbits	Hydrogel-infused scaffolds	[71]
Alg; m-dECMs (3DP-PCL)	Ca^2+^ Crosslinking	MFCs	STS	Cells; rats; rabbits	Hydrogel-infused scaffolds	[62]
GelMA; Ag (3DP-PCL)	Photo-crosslinking; H-bonds	MFCs	None	Cells	Hydrogel-infused scaffolds	[43]
m-dECMs; GelMA (3DP-PCL)	Photo-crosslinking	None	Apt19s; TGF-β3@MPs; CTGF@NPs	Cells*; rats; rabbits	Hydrogel-infused scaffolds	[134]

Cells*: herein, the cells are only used by the researchers for biocompatible evaluation, which is not delivered by hydrogels. In vitro explants^#^: in vitro explants indicate that the meniscus tissues are co-cultured with hydrogels in vitro by the researchers. KGN: kartogenin; DS: diclofenac sodium; NLPs: nanoliposomes; ADA: alginate dialdehyde; MFCs: meniscus fibrochondrocytes; TA-HA: tyramine-modified hyaluronic acid; FB: fibrinogen; PEO: polyethylene oxide; H-bonds: hydrogen bonds; Alg: alginate; GelMA: gelatin methacrylate; CNF: cellulose nanofiber; F127DA: F127 diacrylate; CS-NHS: n-hydroxysuccinimide (NHS)-modified chondroitin sulfate; BM: bone marrow; β-GP: β-glycerophosphate; m-dECMs: meniscus decellularized extracellular matrices; ACs: articular chondrocytes. PEG-CHO: polyethylene glycol aldehyde; Chi: chitosan; MSCs: mesenchymal stem cells; TGF-β1/3: transforming growth factor β-1/3; SIM: simvastatin; FB: fibrinogen; CTGF: connective tissue growth factor; CD44: cluster of differentiation; PRP: platelet-rich plasma; Wnt5a: wingless-type MMTV integration site family member 5a; 3DP-PCL: three-dimensionally printed polycaprolactone; SF: silk fibroin; L7 peptide: LTHPRWP peptide; PEG-NHS: polyethylene glycol NHS-ester; PEG-NH_2_: polyethylene glycol amine; Ac2-26: annexin-1 mimetic peptide; MPs: microspheres; STS: sodium tanshinone IIA sulfonate; Ag: agarose; NPs: nanospheres; Apt19s: aptamer-19s.

## Data Availability

Not applicable.
